# Analysis of gait characteristics to evaluate injury risk after high-intensity exercise, focusing on canoe athletes: a preliminary study

**DOI:** 10.3389/fspor.2025.1652610

**Published:** 2025-10-30

**Authors:** Seoungeun Kim, Minji Son, Seungmin Shin, Taewan Kim, Seungjun Choi

**Affiliations:** ^1^ Research Institute, JEIOS Inc., Busan, Republic of Korea; ^2^ Center for Sports Science in JeonNam, Jangheung County, Jeollanam-do, Republic of Korea; ^3^ Department of Sport Sciences, Kyungsung University, Busan, Republic of Korea

**Keywords:** high-intensity exercise, gait analysis, muscle fatigue, injury risk prediction, canoe

## Abstract

**Objective:**

High-intensity exercise can alter gait characteristics in canoe athletes, potentially affecting performance and increasing injury risk due to muscle fatigue. This study aimed to analyse gait parameters before and after high-intensity exercise to identify fatigue-related injury risk factors.

**Methods:**

Twelve canoe athletes participated. After a brief treadmill acclimation (30–60 s), gait was assessed at three walking speeds: slow (80%), normal (100%), and fast (120%) of preferred speed—for 1 min each. An IMU based shoe-type data logger captured gait data immediately before and after a 30 s Wingate Anaerobic Test.

**Results:**

Significant changes were found in 20 gait parameters. Post-exercise, cadence, stride/step length, single/double support time, time of toe off, ankle ROM (dorsiflexion/plantarflexion, inversion/eversion), and centre of gravity (COG) displacement and velocity in X and Y directions increased. In contrast, COG displacement, velocity, and acceleration in the Z direction decreased.

**Conclusion:**

Gait analysis at slow speed after high-intensity effort highlights the importance of monitoring biomechanical and spatiotemporal changes. Detecting compensatory gait adjustments post-exercise may enable early identification of fatigue-related injury risks, supporting preventive strategies for canoe athletes.

## 1 Introduction

High-intensity exercise is a pivotal component of elite athletic performance, but it considerably increases vulnerability to injuries. Exercises such as sprints or repetitive high-intensity workouts are strongly correlated with lower limb damage (1). This association is primarily driven by acute muscle trauma affecting groups such as the hamstrings, quadriceps, adductors, and calf muscles (2). These injury factors are intrinsically linked to muscle fatigue, which adversely affects neural postural control and increases the risk of injury (3). Additionally, high-intensity exercise can induce changes in the brain state, thereby leading to diminished cognitive function, which significantly affects postural stability and gait patterns (4, 5). Neural, muscular, and joint factors collectively influence injury risk considerably (6).

For example, canoeing increases the risk of sports injuries because of the substantial physical demands it places on the body (7). High-intensity interval exercise can enhance endurance, aerobic and anaerobic capacities, and paddling efficiency of canoeists (8). However, this training intensity can also lead to cellular stress and muscle damage (9). Canoeists require high levels of upper-body strength and generate power from a seated position with extended legs, making lower-body strength crucial (10–12). Strain injuries are most common among canoeists, accounting for 15%–30% of sports injuries annually. Surveys indicate that shoulder injuries are the most prevalent, followed by knee and lower back injuries (13, 14). Factors such as skill level, weather conditions, and the competitive nature of sports contribute to thigh and knee injuries (15).

Recent studies have increasingly focused on predicting these injury factors. Despite extensive efforts to predict sports injuries, inherent limitations exist in identifying these factors (16). Injury prediction is one of the most challenging issues in sports and is critical for injury prevention. Identifying predictive factors is essential (17, 18). Wearable devices such as inertial measurement units (IMUs) have recently been utilised to identify lower limb injury factors, with research focusing on predicting injury risk by using spatiotemporal gait variables (19–24). Spatiotemporal gait variables such as gait speed, cadence, stride length, and variability are critical for predicting injuries (25). Although canoeing is performed in a seated posture and is predominantly upper-body/trunk-dominant, lower-limb force and postural control contribute meaningfully to stroke mechanics: restricting leg drive reduces mean paddle-stroke force and kayak speed, and leg-push forces scale with velocity (26, 27). On-water EMG and kinematic evidence links trunk rotation and abdominal activity with kayak velocity, and prospective data in sprint kayaking show a high burden of upper-limb and trunk injuries (28, 29). Against this backdrop, we used gait analysis as a pragmatic, indirect probe of whole-body neuromuscular fatigue that may affect postural stability and secondary loading relevant to canoe performance, while acknowledging that it is not a sport-specific paddling assessment. Accordingly, this preliminary study used shoe-mounted IMUs to quantify spatiotemporal and kinematic gait parameters at 80%, 100%, and 120% of preferred speed immediately before and after a high-intensity anaerobic effort in canoe athletes, to characterise fatigue-related adaptations that may support early identification of injury risk. Our outcome selection is supported by wearable/IMU literature showing the sensitivity of cadence, stride parameters, and support times to experimentally induced fatigue (20, 21) and by established speed-tier protocols (25), and is consistent with canoe performance evidence emphasising trunk function and upper-body loading (11, 13, 15).

This preliminary study aimed to, first, test whether high-intensity anaerobic exercise elicits measurable changes in fatigue-sensitive gait variables in canoe athletes, particularly at slow speed; and second, explore which variables show the largest standardized pre-post changes as candidate screening markers of neuromuscular fatigue and balance compromise, while explicitly not inferring direct canoe-injury mechanisms from gait alone.

## 2 Materials and methods

### 2.1 Study design and data collection

This study recruited 12 canoe athletes from region C, consisting of 5 athletes from sports high schools and 7 general athletes. The “general athletes” group comprised national and regional representative-level athletes with more than 7 years of canoe training experience. Among them, the general group included 3 males and 4 females, while the sports high school group consisted of 5 males. The detailed gender distribution for each group is presented in Table 1.

**Table 1:** Physical characteristics.

Group	Sex	Sub	Age (y)	Height (cm)	Weight (kg)	Muscle Mass (kg)	Body Fat (%)	Skeletal Muscle Mass (kg)
H	Male	5	18.20 ± .84	170.40 ± 4.72	69.60 ± 10.69	55.44 ± 7.73	13.36 ± 4.73	32.40 ± 5.10
G	Male	3	26.33 ± 6.51	175.07 ± 6.17	80.00 ± 4.36	64.90 ± 2.65	15.13 ± 2.94	39.67 ± 1.46
	Female	4	25.00 ± 4.08	167.35 ± 2.40	62.50 ± 2.38	47.15 ± 1.79	22.28 ± 2.47	27.85 ± 1.13
Cohen's d			−1.966	−0.048	−0.039	0.076	−1.27	−0.087
Total		12	22.50 ± 5.21	170.55 ± 5.08	69.83 ± 9.71	55.04 ± 8.55	16.77 ± 5.33	32.70 ± 5.65

H, high school division; G, general division.

All participants provided written informed consent after receiving a full explanation of the study's purpose, procedures, and potential risks (e.g., fatigue, discomfort). For the two participants under 18 years of age, additional consent was obtained from their legal guardians. The consent form also described data anonymization procedures and confirmed authorization for the publication of anonymized results. The physical characteristics of the participants, categorized by group and sex, are provided in Table 1. Body composition was measured using an InBody 770 device (InBody Co., Ltd., Seoul, Korea). The study protocol was approved by the Institutional Review Board (Approval No. KSU-24-03-003). If participants experienced discomfort, pain, psychological distress, or significant changes during the protocol, the experiment was immediately terminated to ensure their safety.

The relatively small and heterogeneous sample size (*n* = 12) is acknowledged as a core limitation, restricting the generalisability of the findings. However, considering the rarity of elite and specialized athlete populations, this sample size is comparable to that reported in previous pilot studies. To enhance the reliability of interpretation, effect sizes (Cohen's d) were additionally calculated and reported.

Dynamic gait characteristics were assessed using a device with IMUs attached to shoes to measure the gait at various speeds (Motioncore; JEIOS, Busan, Korea). This equipment has a reliability of >98%, compared with that of video equipment by Vicon (Oxford, England). Based on the collected anthropometric data, the body was modelled into parts, such as the head, trunk, arms, and legs, to estimate the moment of inertia for each part. The centre of gravity (COG) was calculated by considering the relative position of each part. The base of support (BOS) was calculated by measuring the positions of the left and right feet during double support in the stance phase of gait. Therefore, gait analysis was categorised into spatiotemporal and kinematic parameters. The spatiotemporal parameters included cadence, stride length, step length, single support time, and double support time, which are crucial for evaluating gait efficiency and stability. Kinematic parameters included the range of motion (ROM) in various planes and the displacement and velocity of the COG, which helps in understanding biomechanical adaptations to high-intensity exercise (30–34).

**Figure 1:**
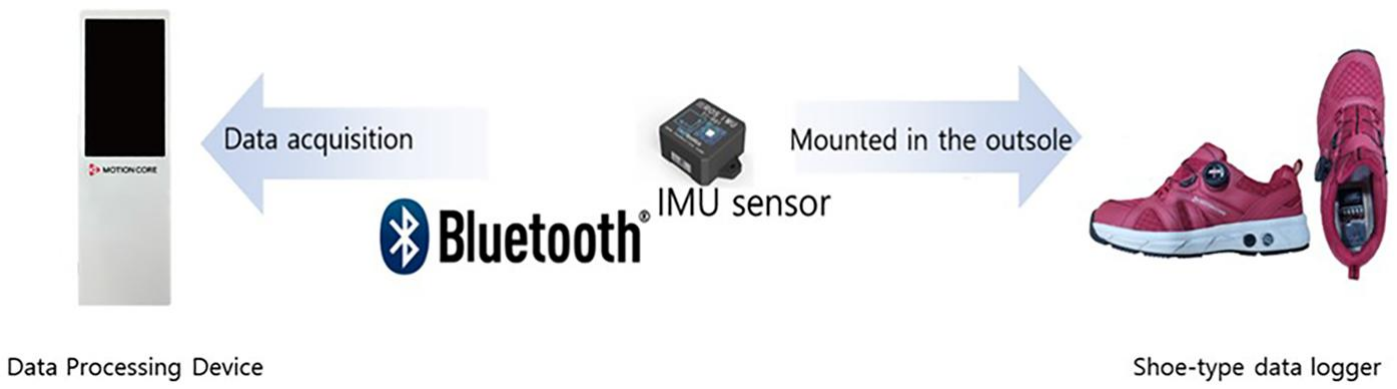
Shoe-type IMU based gait analysis system.

Participants first underwent a treadmill acclimation phase, during which they gradually adjusted the treadmill speed until identifying their most natural walking pace. This speed was sustained for 30–60 s and defined as the preferred walking speed. Based on this, gait was assessed at slow (80%), normal (100%), and fast (120%) of the preferred speed, increments that have been widely adopted in gait studies to examine variability and biomechanical responses (35–38). The order of speed conditions was fixed (slow → normal → fast), with a 30-s seated rest interval between each trial to minimize fatigue bias. The same procedure was applied both before and after the Wingate anaerobic test (Figure 1).

The anaerobic power test was conducted using the Wingate Anaerobic Test, a high-intensity exercise involving 30 s of maximal effort cycling that is widely used to evaluate anaerobic capacity by assessing physiological responses such as lactate concentration and heart rate (39). The protocol followed the guidelines of the Korea Institute of Sport Science (KISS), which are the official standards for physical fitness testing of the Korean national team, with a resistance corresponding to 7.5% of body mass for male participants and 5% for female participants (39). During the test, participants were instructed to maintain a seated position with the trunk leaning slightly forward and both hands firmly gripping the handlebars. This posture was selected to ensure consistent lower-limb loading and to simulate the trunk–leg coordination required in canoe paddling. The primary assessment parameters included peak power and mean power, both expressed relative to body mass (W/kg). Additionally, capillary blood lactate concentration was measured immediately after exercise, and participants' heart rate was continuously monitored using a Polar armband (Verity Sense, Wrocław, Poland).

Blood samples were meticulously collected from the fingertip after disinfecting the skin with 75% alcohol. The first drop of blood was discarded, and subsequent drops were collected using a capillary tube while carefully preventing bubble formation. Samples were immediately transferred into a microtest tube containing a lactate-haemolysing solution for analysis. Blood sampling was conducted at rest and at 5, 10, and 15 min after the anaerobic power test. The blood lactate concentrations of the collected samples were analysed using a lactate analyser (Biosen C-line Lactate Analyser; EKF Diagnostics, Barleben, Germany). The analyser was calibrated daily using standard lactate solutions (5 and 10 mmol/L), with an acceptable measurement error of less than 5%. The resting, peak, and maximum heart rates were measured using a Polar heart rate monitor (Verity Sense) before and after the anaerobic power test.

**Figure 2:**
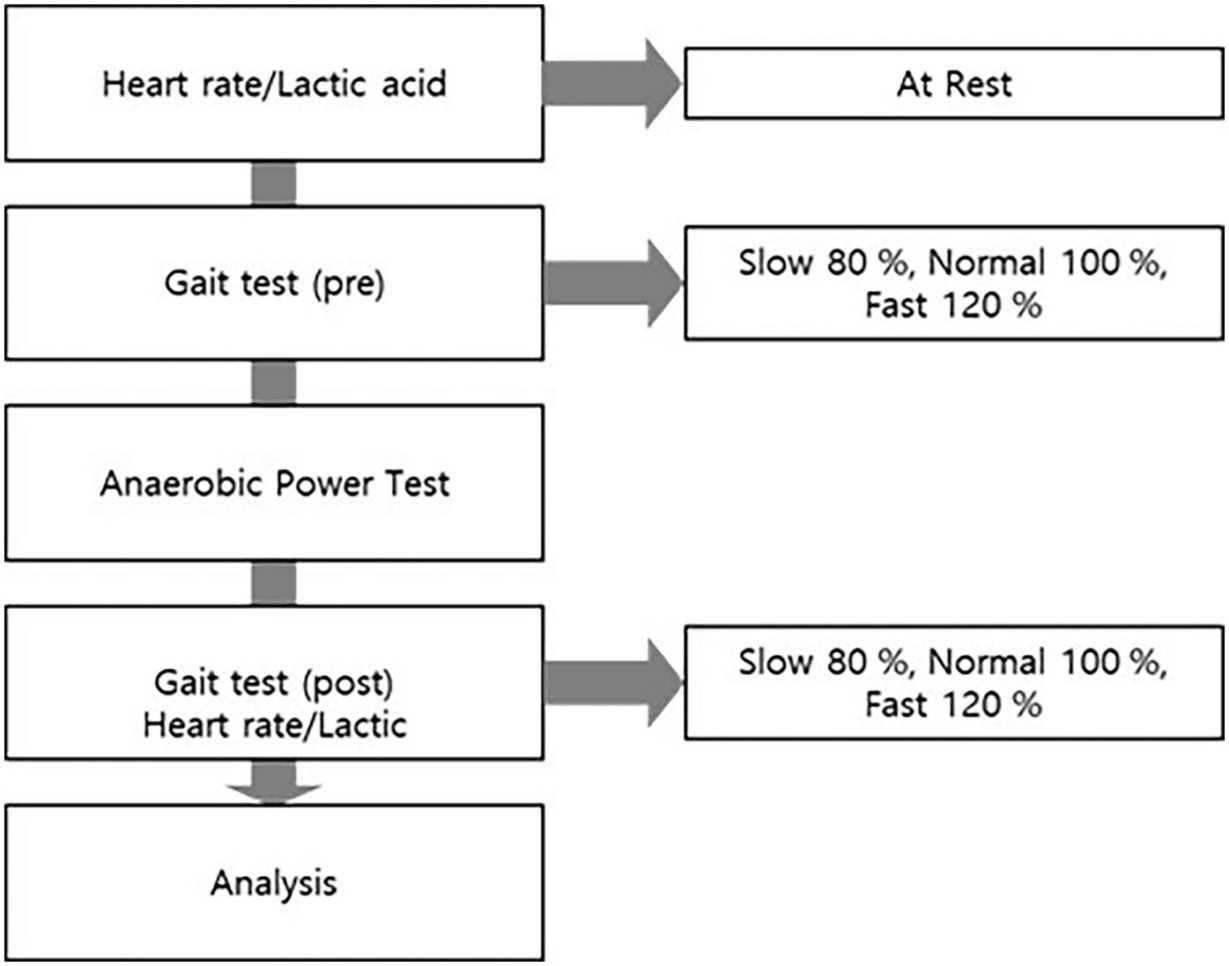
Research flowchart.

The participants were required to maintain a fasting state for 10 h before the test, arrive at the laboratory 30 min before the experiment, and rest in the supine position for the measurement of their resting heart rate. Blood samples for lactate analysis were collected at rest. After blood sampling, the participants were acclimated to the treadmill for 30–60 s to determine their preferred speed. Gait was then measured for 3 min at slow speed (80%), normal speed (100%), and fast speed (120%), with a 30 s rest interval between each speed. Immediately after the anaerobic power test, the maximum and peak heart rates were recorded, and gait characteristics were conducted for each 1 min interval under the same speed conditions. Finally, blood sampling for lactate analysis was conducted 15 min after the test (Figure 2).

### 2.2 Equity, diversity, and inclusion statement

The research team consists of researchers from diverse academic backgrounds, including biomechanics, sports science, and data analysis. Both early-career and experienced researchers collaborate to maintain a balanced approach. The team ensures gender balance, including female researchers, to incorporate a variety of perspectives in research design and result interpretation.

### 2.3 Statistical analysis

All statistical analyses were conducted using SPSS version 23.0 (IBM Corp., Armonk, NY, USA). Normality of the data was assessed using the Shapiro–Wilk test. As most variables did not meet the assumption of normality, non-parametric tests were employed. To examine the effect of walking speed (slow, normal, fast), the Friedman test was conducted, and effect sizes were reported using Kendall's W. *post hoc* comparisons between speed conditions were performed with the Wilcoxon signed-rank test, with Bonferroni correction applied to adjust the significance threshold (α = 0.05/3 = 0.0167). To analyze the effect of high-intensity exercise (pre vs. post), the Wilcoxon signed-rank test was applied separately at each walking speed. Effect sizes were calculated as r = Z/√N, a non-parametric effect size measure for Wilcoxon tests. Here, Z is the Z-score from the test statistic, and N is the number of non-zero paired observations. The significance level was set at α = 0.05, with Bonferroni-adjusted thresholds applied for *post hoc* comparisons.

## 3 Results

### 3.1 Exercise intensity

The results of the anaerobic power test indicated that the average power (in watts) was 396.57 W, and the peak power was 521.53 W. The average power per kilogram was 5.55 W/kg, and the peak power per kilogram was 7.34 W/kg, which aligns with the findings of previous studies (39). Lactate is a crucial indicator of exercise physiology and varies with exercise intensity. Lactate is predominantly produced during anaerobic exercise, and its concentration increases with exercise intensity, potentially exceeding 8 mM/L during maximal-intensity exercise (40). In this study, the mean lactate concentration was 11.90 mM/L, indicating maximal-intensity exercise. The anaerobic threshold (AT) refers to the exercise intensity at which lactate begins to accumulate rapidly, typically occurring at 50%–80% of maximal oxygen uptake. Oxygen uptake is proportional to the heart rate; therefore, 75% of the maximal oxygen uptake corresponds to approximately 85% of the maximal heart rate. The results of this study showed a peak heart rate of 78.87%, confirming that the exercise intensity in this study was high-intensity (Table 2).

**Table 2:** Exercise intensity.

Heart Rate	Resting (Hr)	Maximum (Hr)		HRmax (%)	
	67.08 ± 5.09	153.58 ± 11.16		79.87 ± 5.48	
Anaerobic Power Test	Average power (W)	Peak power (W)		Average power (W/kg)	Peak power (W/kg)
	396.57 ± 140.42	521.53 ± 179.40		5.55 ± 1.40	7.34 ± 1.94
Lactate Test	Stability (mm/L)	Immediate (mm/L)	5 min (mm/L)	10 min (mm/L)	15 min (mm/L)
	1.45 ± 0.42	6.50 ± 1.46	11.90 ± 1.16	11.74 ± 1.36	10.71 ± 1.23

HRmax, maximum heart rate.

### 3.2 Spatiotemporal analysis results

The analysis of spatiotemporal parameters before and after high-intensity exercise at slow, normal, and fast speeds showed that all variables, except gait asymmetry (GA), differed significantly among speeds (all *χ*², *p* < .05; Kendall's W = 0.92–1.00; Table 3). *post-hoc* tests confirmed significant pairwise differences across speeds (a, b, c) for cadence, stride length, step length, single support, double support, and toe-off timing.

**Table 3:** Analysis results of spatiotemporal parameters.

Variable	Pre/post	Slow	Normal	Fast	*χ²*	Effect size (*W*)	Post-hoc (speed)
Cadence (steps/min)	Pre	82.50 ± 6.60	93.08 ± 7.19	100.92 ± 5.92	24.00*	1.00	a,b,c
	Post	87.75 ± 6.55	94.50 ± 4.93	101.00 ± 5.88	24.00*	1.00	a,b,c
	*z-score* (Effect size)	2.94* r *=* 0.85	1.29 *r* *=* 0.37	0.32 *r* *=* 0.09			
NormSTL (%)	Pre	0.73 ± 0.09	0.81 ± 0.07	0.87 ± 0.07	24.00*	1.00	a,b,c
	Post	0.72 ± 0.10	0.81 ± 0.08	0.87 ± 0.07	24.00*	1.00	a,b,c
	*z-*score (Effect size)	0.86 *r* *=* 0.25	0.08 *r* *=* 0.02	0.24 *r* *=* 0.07			
Gait Asymmetry (%)	Pre	1.59 ± 1.14	1.94 ± 1.30	1.52 ± 1.23	3.50	0.15	ns
	Post	1.97 ± 1.71	2.24 ± 1.03	1.55 ± 1.46	2.17	0.09	ns
	*z-*score (Effect size)	0.31 *r* *=* 0.09	0.47 *r* *=* 0.14	0.24 *r* *=* 0.07			
Left Stride Length (m)	Pre	1.04 ± 0.12	1.16 ± 0.11	1.29 ± 0.11	22.17*	0.92	a,b,c
	Post	0.98 ± 0.09	1.14 ± 0.10	1.28 ± 0.11	24.00*	1.00	a,b,c
	*z-*score (Effect size)	2.98* r *=* 0.86	2.04* r *=* 0.59	1.73 *r* *=* 0.50			
Right Stride Length (m)	Pre	1.04 ± 0.12	1.16 ± 0.11	1.29 ± 0.11	22.17*	0.92	a,b,c
	Post	0.98 ± 0.09	1.14 ± 0.10	1.27 ± 0.11	24.00*	1.00	a,b,c
	*z-*score (Effect size)	2.98* r *=* 0.86	2.04* r *=* 0.59	1.73 *r* *=* 0.50			
Left Step Length (m)	Pre	0.52 ± 0.06	0.58 ± 0.05	0.64 ± 0.06	22.17*	0.92	a,b,c
	Post	0.49 ± 0.05	0.57 ± 0.05	0.64 ± 0.06	24.00*	1.00	a,b,c
	*z-*score (Effect size)	2.98* r *=* 0.86	2.04* r *=* 0.59	1.73 *r* *=* 0.50			
Right Step Length (m)	Pre	0.52 ± 0.06	0.58 ± 0.05	0.64 ± 0.06	22.17*	0.92	a,b,c
	Post	0.49 ± 0.05	0.57 ± 0.05	0.64 ± 0.06	24.00*	1.00	a,b,c
	*z-*score (Effect size)	2.98* r *=* 0.86	2.04* r *=* 0.59	1.73 *r* *=* 0.50			
Left Single Support (%)	Pre	38.24 ± 1.47	39.86 ± 1.10	41.14 ± 1.21	22.17*	0.92	a,b,c
	Post	37.52 ± 1.70	39.28 ± 1.00	40.83 ± 1.15	24.00*	1.00	a,b,c
	*z-*score (Effect size)	2.35* r *=* 0.68	2.43* r *=* 0.70	1.96* r *=* 0.57			
Right Single Support (%)	Pre	38.13 ± 1.69	39.71 ± 1.38	40.98 ± 1.43	24.00*	1.00	a,b,c
	Post	37.20 ± 1.97	39.30 ± 1.45	40.70 ± 1.22	24.00*	1.00	a,b,c
	*z-*score (Effect size)	2.35* r *=* 0.68	2.12* r *=* 0.61	2.12* r *=* 0.61			
Left Double Support (%)	Pre	38.24 ± 1.47	39.86 ± 1.10	41.14 ± 1.21	22.17*	0.92	a,b,c
	Post	37.52 ± 1.70	39.28 ± 1.00	40.83 ± 1.15	24.00*	1.00	a,b,c
	*z-*score (Effect size)	2.35* r *=* 0.68	2.43* r *=* 0.70	1.96* r *=* 0.57			
Right Double Support (%)	Pre	23.53 ± 3.09	20.35 ± 2.35	17.80 ± 2.53	22.17*	0.92	a,b,c
	Post	25.18 ± 3.56	21.35 ± 2.31	18.38 ± 2.22	24.00*	1.00	a,b,c
	*z-*score (Effect size)	2.67* r *=* 0.77	3.06* r *=* 0.88	2.27* r *=* 0.66			
Left Time of Toe off (%)	Pre	61.93 ± 1.64	60.37 ± 1.34	59.08 ± 1.39	24.00*	1.00	a,b,c
	Post	62.80 ± 1.95	60.77 ± 1.43	59.38 ± 1.20	24.00*	1.00	a,b,c
	*z-*score (Effect size)	2.35* r *=* 0.68	2.12* r *=* 0.61	2.04* r *=* 0.59			
Right Time of Toe off (%)	Pre	61.60 ± 1.49	59.99 ± 1.15	58.72 ± 1.22	22.17*	0.92	a,b,c
	Post	62.35 ± 1.70	60.59 ± 1.02	59.02 ± 1.18	24.00*	1.00	a,b,c
	*z-*score (Effect size)	2.59* r *=* 0.75	2.67* r *=* 0.77	1.88 *r* *=* 0.54			

Comparison of spatiotemporal gait parameters before and after high-intensity exercise at three walking speeds (slow, normal, fast). Data are presented as mean ± standard deviation (SD). Differences among walking speeds were examined using the Friedman test (*χ*²) with effect sizes reported as Kendall's W. *post-hoc* pairwise comparisons were performed with the Wilcoxon signed-rank test, with Bonferroni correction applied (adjusted α = 0.0167; a = slow vs. normal, b = slow vs. fast, c = normal vs. fast). Pre–post comparisons within each speed condition are reported as Wilcoxon *z-*scores with corresponding effect sizes (*r*) and *p*-values.

* *p* < .05; ns, not significant.

Pre–post comparisons within each speed condition revealed that cadence, double support, and toe-off timing increased significantly after exercise (all *p* < .05, *r* = 0.59–0.77), whereas normalized stride length, stride length, step length, and single support decreased significantly (all *p* < .05, *r* = 0.57–0.86). GA showed no significant exercise effect. These changes were most pronounced at slow walking speed, indicating compensatory temporal and spatial adjustments in response to exercise-induced fatigue.

### 3.3 Kinematic analysis results

The analysis of kinematic parameters revealed significant speed effects for most ankle ROM and COG measures, except for inver-eversion ROM, add-abduction ROM, and COG acceleration Y (Table 4). Friedman tests showed large effect sizes across speeds (*χ²*, *p* < .05; *W* = 0.26–1.00). *post-hoc* comparisons indicated that slow vs. fast and normal vs. fast conditions accounted for most differences.

**Table 4:** Analysis results of kinematic parameters.

Variable	Pre/post	Slow	Normal	Fast	*χ²*	Effect size (*W*)	Post-hoc (speed)
Left ROM Inver/Ever (deg)	Pre	−3.85 ± 2.31	−4.58 ± 2.61	−4.81 ± 2.66	12.17*	0.51	a,b
	Post	−4.19 ± 2.18	−4.69 ± 2.51	−5.08 ± 2.79	8.67*	0.36	ns
	*z-*score (Effect size)	1.26 *r* *=* 0.36	0.16 *r* *=* 0.05	0.47 *r* *=* 0.14			
Right ROM Inver/Ever (deg)	Pre	−4.08 ± 3.90	−5.19 ± 4.47	−5.12 ± 4.28	1.17	0.42	ns
	Post	−5.29 ± 4.25	−5.95 ± 5.15	−5.93 ± 5.16	3.50	0.09	ns
	*z-*score (Effect size)	2.98* r *=* 0.86	2.04* r *=* 0.59	1.73 *r* *=* 0.50			
Left ROM Dorsi/Plantar (deg)	Pre	−10.85 ± 2.76	−12.94 ± 2.50	−14.83 ± 2.70	22.17*	0.92	a,b,c
	Post	−8.73 ± 2.62	−11.65 ± 2.74	−14.06 ± 2.52	24.00*	1.00	a,b,c
	*z-*score (Effect size)	2.82* r *=* 0.82	2.12* r *=* 0.61	1.49 *r* *=* 0.43			
Right ROM Dorsi/Plantar (deg)	Pre	−11.26 ± 2.42	−12.93 ± 2.19	−15.09 ± 2.59	22.17*	0.92	a,b,c
	Post	−9.48 ± 2.31	−12.31 ± 2.61	−14.37 ± 2.43	24.00*	1.00	a,b,c
	*z-*score (Effect size)	2.75* r *=* 0.79	0.86 *r* *=* 0.25	1.49 *r* *=* 0.43			
Left ROM Adduct/Abduct (deg)	Pre	0.41 ± 0.70	0.43 ± 0.78	0.60 ± 0.74	6.17*	0.26	c
	Post	0.11 ± 0.81	0.32 ± 0.91	0.34 ± 1.06	6.17	0.26	ns
	*z-*score (Effect size)	2.04* r *=* 0.59	0.16 *r* *=* 0.05	1.18 *r* *=* 0.34			
Right ROM Adduct/Abduct (deg)	Pre	0.34 ± 0.91	0.42 ± 0.94	0.53 ± 1.05	1.17	0.05	ns
	Post	−0.05 ± 1.17	−0.10 ± 1.88	−0.09 ± 2.15	3.50	0.15	ns
	*z-*score (Effect size)	2.82* r *=* 0.82	1.65 *r* *=* 0.48	2.20* r *=* 0.63			
COG Displacement X (m)	Pre	0.09 ± 0.01	0.11 ± 0.01	0.13 ± 0.01	24.00*	1.00	a,b,c
	Post	0.09 ± 0.01	0.11 ± 0.01	0.13 ± 0.01	24.00*	1.00	a,b,c
	*z-*score (Effect size)	2.12* r *=* 0.61	2.04* r *=* 0.59	0.94 *r* *=* 0.27			
COG Displacement Y (m)	Pre	0.03 ± 0.01	0.03 ± 0.01	0.04 ± 0.01	24.00*	1.00	a,b,c
	Post	0.03 ± 0.01	0.04 ± 0.01	0.04 ± 0.01	20.17*	0.84	a,b,c
	*z-*score (Effect size)	3.06* r *=* 0.88	2.04* r *=* 0.59	1.33 *r* *=* 0.38			
COG Displacement Z (m)	Pre	0.03 ± 0.01	0.03 ± 0.00	0.02 ± 0.00	22.17*	0.92	a,b,c
	Post	0.03 ± 0.01	0.03 ± 0.01	0.02 ± 0.00	15.17*	0.63	a,b,c
	*z-*score (Effect size)	1.80 *r* *=* 0.52	0.00 *r* *=* 0.00	0.82 *r* *=* 0.24			
COG Velocity X (m/s)	Pre	0.90 ± 0.12	1.12 ± 0.13	1.30 ± 0.12	24.00*	1.00	a,b,c
	Post	0.94 ± 0.15	1.14 ± 0.14	1.31 ± 0.12	24.00*	1.00	a,b,c
	*z-*score (Effect size)	2.12* r *=* 0.61	2.04* r *=* 0.59	0.94 *r* *=* 0.27			
COG Velocity Y (m/s)	Pre	0.26 ± 0.07	0.34 ± 0.10	0.38 ± 0.11	24.00*	1.00	a,b,c
	Post	0.31 ± 0.10	0.36 ± 0.12	0.39 ± 0.12	20.17*	0.84	a,b,c
	*z-*score (Effect size)	3.06* r *=* 0.88	2.04* r *=* 0.59	1.41 *r* *=* 0.41			
COG Velocity Z (m/s)	Pre	0.35 ± 0.09	0.25 ± 0.04	0.21 ± 0.03	22.17*	0.92	a,b,c
	Post	0.30 ± 0.08	0.25 ± 0.06	0.20 ± 0.04	15.17*	0.63	a,b,c
	*z-*score (Effect size)	1.80 *r* *=* 0.52	0.08 *r* *=* 0.02	0.86 *r* *=* 0.25			
COG Acceleration X (m/s^2^)	Pre	0.35 ± 0.09	0.25 ± 0.04	0.21 ± 0.03	22.17*	0.92	a,b,c
	Post	0.30 ± 0.08	0.25 ± 0.06	0.20 ± 0.04	15.17*	0.63	a,b,c
	*z-*score (Effect size)	1.80 *r* *=* 0.52	0.08 *r* *=* 0.02	0.86 *r* *=* 0.25			
COG Acceleration Y (m/s^2^)	Pre	0.13 ± 0.04	0.11 ± 0.03	0.10 ± 0.03	8.00*	0.33	a,b
	Post	0.13 ± 0.05	0.12 ± 0.04	0.10 ± 0.04	6.50*	0.27	ns
	*z-*score (Effect size)	0.78 *r* *=* 0.23	0.08 *r* *=* 0.02	0.55 *r* *=* 0.16			
COG Acceleration Z (m/s^2^)	Pre	0.29 ± 0.07	0.20 ± 0.04	0.18 ± 0.04	16.17*	0.67	a,b
	Post	0.24 ± 0.07	0.19 ± 0.04	0.17 ± 0.04	10.67*	0.44	b
	*z-*score (Effect size)	2.35* r *=* 0.68	1.18 *r* *=* 0.34	1.47 *r* *=* 0.42			

Comparison of ankle joint range of motion (ROM) before and after high-intensity exercise at three walking speeds (slow, normal, fast). Data are presented as mean ± standard deviation (SD). Differences among walking speeds were examined using the Friedman test (*χ*²), with effect sizes reported as Kendall's W. *post-hoc* pairwise comparisons were performed with the Wilcoxon signed-rank test, with Bonferroni correction applied (adjusted α = 0.0167; a = slow vs. normal, b = slow vs. fast, c = normal vs. fast). Pre–post comparisons within each speed condition are reported as Wilcoxon *z-*scores with corresponding effect sizes (*r*) and *p*-values.

* *p* < .05; ns, not significant.

Pre–post comparisons demonstrated significant reductions in ankle dorsi-plantar flexion ROM (*p* < .05, *r* = 0.61–0.82) and decreases in COG velocity and acceleration along the X and Z axes (*p* < .05, *r* = 0.52–0.68). Conversely, COG displacement Y increased significantly after exercise (*p* < .05, *r* = 0.88). Other variables, such as inver-eversion ROM, did not show significant exercise effects. Collectively, these results indicate that high-intensity exercise alters both joint ROM and COG dynamics, with the most pronounced fatigue-related changes observed at slow speeds.

## 4 Discussion

To investigate fatigue-related adaptations in gait, we analysed three walking speeds: slow (80%), normal (100%), and fast (120%) of preferred speed—which are commonly used to examine biomechanical and spatiotemporal responses (36–39, 41). Slow speed accentuates stability constraints and can markedly alter gait mechanics (42). At these slower speeds, stride duration and joint coordination change significantly, reducing limb clearance, and the temporal order of maximum joint impact reverses (43). Employing differentiated gait speeds therefore allowed us to evaluate how the interaction of biomechanical adjustments and cognitive control contributes to injury risk.

Cadence can increase as a compensatory mechanism to maintain stability and forward momentum, which is influenced by fatigue (44). In canoe-specific biomechanics, the increase in cadence observed may represent a compensatory strategy when trunk–limb coordination is impaired. Canoe paddling requires propulsion through upper-body movements while maintaining trunk stability, and when trunk control is weakened, athletes may reduce stride length and increase step frequency to preserve balance. This is consistent with previous research showing that fatigue-related torso acceleration during gait affects cadence (45).

NormSTL reflects the relationship between stride length and individual height, providing a sensitive index of gait patterns. Single support requires a high level of dynamic balance, making it more challenging than double support (46). Its decrease is linked to negative central nervous system effects on motor control (47). For canoe athletes, reduced single support and stride length may indicate impaired balance after fatigue. Although not direct indicators of canoe-specific injury mechanisms, they may serve as indirect markers of systemic fatigue influencing paddling mechanics. This pragmatic use of gait analysis acknowledges its environmental limitations while offering a feasible proxy of neuromuscular fatigue.

An increase in double support indicates a longer support phase for both feet. This may seem counterintuitive compared with a decrease in single support; however, interpreting it alone can be problematic given the complexity of gait (48). In this study, the post-exercise increase in double support may have been due to reduced gait rhythm caused by physical decline (49).

The time of toe off influences gait initiation by supporting postural control and propulsion (50). In this study, toe-off time increased, suggesting prolonged muscle tension, higher energy consumption, and reduced gait efficiency. From a canoe-specific perspective, this resembles sustained trunk–leg force transmission during paddling. While gait analysis cannot replicate paddling biomechanics, these changes may indicate secondary loading pathways that elevate injury risk.

The kinematic analysis suggested that walking with 10° adduction can increase lateral muscle co-contraction, thereby reducing medial knee load and the knee adduction moment (51). Increases in COG displacement and velocity indicate that deterioration of postural control due to fatigue can affect proprioception, sensory processing, and force generation (52). In canoe-specific terms, reduced vertical COG oscillation may compromise natural shock absorption, increasing joint load and tendon stress. Although not direct sport-specific injury markers, these changes align with prior canoeing and rowing studies linking trunk instability and altered lower-limb mechanics to overuse injuries.

Importantly, this study is among the first to apply gait analysis to canoe athletes, and no prior research has validated this approach in seated sports. The lack of comparable studies is acknowledged; however, evidence from other athletes shows that fatigue-induced gait adaptations reflect systemic neuromuscular impairment, justifying its exploratory use. Future investigations should integrate trunk electromyography, seated balance assessments, or simulated paddling motion capture to establish stronger sport-specific links.

Overall, analysis revealed significant differences in 20 spatiotemporal and kinematic parameters, including cadence, stride length, step length, single/double support, toe off time, ankle ROMs, and COG variables, which were particularly pronounced at slow speeds.

## 5 Limitations

One limitation of this study is the small sample size of 12 participants, which may have affected the generalisability of the findings. Future research should include a larger and more diverse sample of athletes to validate these results. The study also focused only on a specific subset of spatiotemporal and kinematic parameters. Expanding the scope to other variables could provide a more comprehensive understanding of gait mechanics and injury risk.

More importantly, although canoeing is a seated sport primarily involving upper-body and trunk mechanics, gait analysis was used here as an indirect proxy of systemic fatigue and balance-related adaptations. This methodological incongruity is a central limitation, as gait variables such as stride length or dorsiflexion may not directly reflect canoe-specific injury mechanisms.

To address this, future investigations should integrate complementary assessments, including trunk electromyography (EMG), seated balance tests, and simulated paddling motion capture. Combining these with gait analysis may provide a more holistic understanding of fatigue and injury risk. Additionally, applying mixed-effects models to repeated measurements could better capture both inter- and intra-athlete variability, thereby improving validity.

Despite these limitations, this study provides foundational data on how high-intensity exercise influences gait characteristics in canoe athletes and highlights critical areas for injury prevention.

## 6 Conclusion

This study confirmed that classifying gait speed as slow (i.e., 80%) is essential for understanding biomechanical and spatiotemporal changes in gait patterns after high-intensity exercise in canoe athletes. As compensatory mechanisms to maintain stability and forward momentum, cadence, double support, toe off time, and COG displacement/velocity (X and Y) increased, whereas stride length, step length, single support, ankle dorsiflexion/plantarflexion ROM, ankle adduction/abduction ROM, and COG vertical and acceleration measures decreased. These changes should be interpreted cautiously, as gait analysis cannot fully capture canoe paddling biomechanics. Nonetheless, the observed fatigue-induced alterations suggest systemic impairments in postural control and motor function that may secondarily influence paddling mechanics and injury risk.

Therefore, gait monitoring—particularly at slower speeds—may serve as a valuable tool for early detection of fatigue-related instability in canoe athletes. To establish stronger sport-specific validity, future research should combine gait analysis with trunk EMG, seated balance, and simulated paddling evaluations.

## 7 Clinical implications

High-intensity exercise induces significant changes in gait, particularly at slower speeds, affecting cadence, stride length, step length, and support times. These changes suggest that fatigue can impair postural control and coordination, increasing injury risk. Although gait analysis is not a sport-specific assessment for canoeing, it provides a practical tool to detect systemic fatigue and balance-related impairments that may indirectly affect paddling mechanics. Reductions in single support and stride length, and increases in cadence and double support, highlight fatigue-induced instability that can compromise performance.

For clinical and training use, coaches and sports scientists should interpret gait data as complementary information rather than direct indicators of canoe-specific injury. Monitoring gait patterns—especially under fatigued conditions and at slower speeds—can help identify athletes at higher risk of instability, prompting preventive strategies.

Future applications should integrate gait analysis with trunk EMG, seated balance testing, and sport-specific paddling evaluations. Such combined approaches could support individualized training adjustments, optimize recovery strategies, and ultimately reduce overuse injuries.

## Data Availability

The original contributions presented in the study are included in the article/Supplementary Material, further inquiries can be directed to the corresponding author.
